# Reconstructing Social Participation in Rural Communities: A Qualitative Study of Community Nursing Practice

**DOI:** 10.7759/cureus.106101

**Published:** 2026-03-30

**Authors:** Ryuichi Ohta, Akiko Yata

**Affiliations:** 1 Community Care, Unnan City Hospital, Unnan, JPN; 2 Family Medicine, Community Nurse Company, Izumo, JPN

**Keywords:** aged, community health nursing, health promotion, qualitative research, rehabilitation, rural population, social isolation, social participation

## Abstract

Introduction

Participation is a central outcome in community-based rehabilitation, particularly in aging societies where social isolation among older adults is increasing. Community nurses have emerged as key facilitators of social participation in community settings. However, the mechanisms through which community nurses reconstruct participation in everyday practice remain insufficiently understood. This study aimed to clarify how community nurses reconstruct and facilitate participation in community-based rehabilitation.

Methods

This retrospective qualitative study analyzed activity logs and reflective practice records documented by three community nurses working in a rural Japanese community between January and December 2025. Thematic analysis following Braun and Clarke's framework was conducted. Participation episodes were identified and analyzed to examine how participation emerged and was facilitated in everyday community practice. Identified activities were also interpreted with reference to the participation concept of the International Classification of Functioning, Disability and Health (ICF).

Results

A total of 134 participation episodes were identified across multiple participation domains, including community life, recreation and leisure, interpersonal relationships, and digital communication. Four themes were identified: (1) creating participation-ready environments, (2) facilitating reciprocal roles and resident empowerment, (3) reconstructing participation through personal narratives and identity, and (4) bridging participation barriers through relational and structural support. Participation emerged as a relational and dynamic process embedded in everyday community interactions.

Conclusions

Community nurses play a critical role in reconstructing participation in community-based rehabilitation by facilitating relational engagement, enabling meaningful social roles, and reducing participation barriers. These findings provide empirical insights into how participation can be operationalized in community practice and highlight the potential of participation-oriented approaches in aging societies.

## Introduction

Population aging has accelerated globally, transforming the social structure of communities and fundamentally altering patterns of human relationships [1,2]. As societies age, traditional forms of social interaction, such as intergenerational cohabitation, neighborhood cohesion, and informal mutual support, have declined, increasing the risk of social isolation among older adults [3]. Social isolation is associated with adverse health outcomes, including increased morbidity, mortality, cognitive decline, and reduced quality of life [4,5]. Consequently, strengthening social connectedness and enhancing social capital have become critical priorities for sustaining healthy and resilient communities in aging societies [6].

Social capital, defined as the networks, norms, and trust that facilitate coordination and cooperation among individuals, plays a central role in promoting health and well-being [7]. However, despite the recognized importance of social capital, some individuals remain socially isolated or are unable to participate in community life, even when opportunities or potential connections exist [8]. This lack of participation is not always due to individual functional limitations but may result from structural barriers, insufficient facilitation, or limited opportunities for engagement [9]. As a result, many communities possess latent resources and assets that could sustain vitality and mutual support, yet these resources are not fully activated or accessible to all community members [10].

In response to these challenges, community nurses (CNs) have emerged in Japan as new actors who facilitate connections between individuals and community resources [11]. CNs engage in community-based activities, build relationships through ongoing dialogue with residents, and support individuals in accessing social opportunities [12,13]. Although not all CNs are formally licensed nurses, they possess nursing-informed competencies, including relational engagement, attentive listening, and contextual understanding of individuals' lived experiences [13]. Through these relational practices, CNs serve as intermediaries, connecting individuals and community resources to enable meaningful social participation [14].

Participation is a core component of health and rehabilitation, as defined by the International Classification of Functioning, Disability and Health (ICF), which conceptualizes participation as involvement in life situations and social roles [15]. The ICF framework emphasizes that health is determined not solely by physical or cognitive function but also by the ability to engage in meaningful social activities and community life [15]. From this perspective, rehabilitation extends beyond restoring individual function to facilitating participation within social and environmental contexts. Increasing participation is therefore essential for maintaining health, promoting well-being, and sustaining community vitality. Community-based activities led by CNs may enhance participation by creating opportunities for engagement, strengthening social relationships, and enabling individuals to reconnect with their communities.

However, despite the growing presence of CNs and their potential to promote participation, the specific mechanisms by which their activities facilitate participation within the ICF framework remain insufficiently understood. In particular, there is limited empirical research examining how CNs operationalize participation in everyday community contexts and how their relational practices contribute to participation-oriented rehabilitation.

Therefore, this study aims to clarify how CNs reconstruct social participation in community settings by qualitatively analyzing activity logs and reflective practice records. By identifying participation episodes and examining how participation is facilitated in everyday community practice, this study seeks to provide empirical insights into the mechanisms through which CNs support participation-oriented community rehabilitation in aging societies.

## Materials and methods

Study design

This study employed a retrospective qualitative study design using document analysis to examine how CNs facilitate participation within community settings. A qualitative descriptive approach was chosen to capture CNs' real-world practices and to explore how participation, as conceptualized in the ICF, was operationalized in everyday community interactions. This approach is appropriate for examining practice-based phenomena where naturally occurring data provide insights into social processes and relational dynamics.

Study setting

This study was conducted in Sarabetsu Village, a rural agricultural community located in Hokkaido, northern Japan. Sarabetsu Village has a population of approximately 3,000 residents and is characterized by a rapidly aging demographic structure, with a high proportion of older adults and increasing risks of social isolation. Like many rural areas in Japan, the community faces challenges related to population aging, geographic dispersion, and limited access to formal healthcare and social support services.

In this setting, three CNs were deployed and embedded within the community to support residents' health and well-being through community-based engagement. These CNs entered the community as active participants rather than external healthcare providers and conducted their activities across various local settings, including community centers, public gathering spaces, local businesses, and residents' homes. Their activities included outreach to residents, facilitation of social interactions, support for community participation, coordination of local resources, and ongoing relational engagement with community members.

Through sustained presence and interaction, CNs functioned as relational facilitators who supported individuals' involvement in meaningful life situations and community activities. Rather than focusing solely on clinical care, their work emphasized enabling participation by connecting residents with social opportunities, fostering interpersonal relationships, and facilitating engagement with existing community resources.

Data source and data collection

The source data consisted of monthly narrative practice reports ("Hyaku-Waku Reports") produced by a team of three CNs working in Sarabetsu Village between January and December 2025. Each monthly report compiled multiple dated entries documenting CN activities across diverse local settings, including community gathering spaces, public facilities, hot spring lobbies, cafés, and residents' homes. These entries were primarily written in free-text narrative form and typically included the date, place, residents involved, context and content of the interaction, actions taken by the CNs, and observational notes regarding residents' responses and engagement.

The records included both descriptive activity logs and reflective practice components. Many entries documented concrete support for participation, such as inviting residents to community activities, facilitating conversation, supporting resident-led initiatives, and assisting with digital tools. Some entries also contained reflective interpretations of how these interactions influenced participation, relationships, and the nurses' understanding of community dynamics. This structure allowed us to analyze not only what activities occurred but also how CNs interpreted participation as an evolving relational process in everyday practice.

Analytical framework

Data were analyzed using thematic analysis following Braun and Clarke's framework, with interpretation informed by the participation concept of the ICF. The ICF defines participation as involvement in life situations, including social interactions, community life, and engagement in meaningful roles. The analysis combined inductive and deductive approaches. Inductive analysis was used to identify patterns and themes emerging from the data without predefined assumptions. Deductive analysis was used to interpret these themes in relation to the ICF participation construct. This combined approach enabled both the empirical description and theoretical interpretation of CN practices.

Identification and quantification of participation episodes and ICF linking

A participation episode was operationally defined as a discrete segment within an activity log or reflective practice record in which a CN facilitated, enabled, or supported a resident's involvement in a life situation corresponding to the ICF participation component. Episodes were delineated based on shifts in the focal activity, participant constellation, or participation-related purpose within a single record. Therefore, more than one participation episode could be extracted from a single activity log when multiple participation-facilitating actions or situations were documented. Episodes were included when the record contained sufficient detail to identify the following: (1) the CN action or relational practice, (2) the resident's actual or intended involvement in a life situation, and (3) a plausible linkage to an ICF participation domain. Descriptions limited to administrative coordination, internal staff communication, or purely observational notes without resident participation were excluded. For example, if one log described a CN first inviting a resident to a community gathering space and later supporting the same resident in using a smartphone application to communicate with others, these were treated as two separate participation episodes because they involved different participation processes and ICF domains.

The first author (RO) reviewed all activity logs and identified participation episodes based on this operational definition. Each episode was then categorized according to its primary activity type and linked to the corresponding ICF participation domains using established ICF linking principles. The second author (AY), who has extensive expertise in CN practice and organizational management, reviewed the episode categorization and ICF linkage. Through iterative discussion and consensus, discrepancies were resolved, and classifications were refined. The number and proportion of participation episodes in each category were calculated to describe the distribution of CN participation-oriented practices. This quantification was used to complement the thematic analysis by providing descriptive context for the range and relative frequency of participation facilitation activities.

Qualitative data analysis procedure

Data were analyzed using thematic analysis following the six-phase framework described by Braun and Clarke, informed by the participation domain of the ICF [16]. Coding was conducted manually by RO without the use of qualitative data analysis software. After repeated reading of the records, RO highlighted meaning units related to participation, facilitation, relational engagement, and barriers to involvement. Initial descriptive codes were assigned inductively to these meaning units and compiled into an analytic coding table. Codes with conceptual overlap were then grouped, compared across records, and iteratively refined into broader categories and candidate themes. AY independently reviewed the coding structure, code definitions, and candidate themes from the perspective of CN practice. Any differences in interpretation were discussed in several rounds of review until a consensus was reached. Rather than calculating inter-rater agreement, discrepancies were resolved through reflexive dialogue to ensure conceptual coherence and contextual validity.

Regarding phase 1 of familiarization with the data, RO repeatedly read all activity logs and reflective practice records to achieve deep immersion in the data and gain a comprehensive understanding of CN activities and interactions within the community. This process enabled the identification of initial patterns related to participation and relational engagement. Regarding phase 2 of initial coding, RO identified meaningful text segments related to participation, social interaction, facilitation, and engagement. Codes captured both CN actions and community members' responses, behaviors, and participation. Coding was conducted inductively, allowing patterns to emerge directly from the data without imposing predefined categories. In phase 3 of candidate theme generation, RO grouped related codes into broader themes based on conceptual similarities and functional relationships. These themes represented patterns in how CNs facilitated participation and enabled engagement in community life. Regarding phase 4 of theme refinement through collaborative review, AY, who has extensive experience in CN education and organizational leadership, reviewed the coding structure and candidate themes. Through iterative discussions between RO and AY, themes were refined to ensure coherence, consistency, and contextual accuracy. This collaborative review process enhanced the credibility and trustworthiness of the analysis by integrating both analytical interpretation and practice-based expertise. Regarding phase 5 of mapping to the ICF participation framework, the refined themes were then interpreted deductively within the participation domain of the ICF. This process enabled the identification of how CN activities contributed to individuals' involvement in life situations, including social interaction, community engagement, and meaningful role participation. Finally, in phase 6 of conceptual synthesis, RO synthesized the themes into a conceptual interpretation of how CNs facilitate and reconstruct participation in community settings. AY provided further feedback on the conceptual interpretation to ensure alignment with CN practice and organizational context. This process resulted in a theoretically informed and empirically grounded model of participation-oriented CN practice.

Rigor, trustworthiness, and reflexivity

To enhance rigor and trustworthiness, this study followed established qualitative research criteria, including credibility, dependability, confirmability, and transferability. Credibility was ensured through prolonged engagement with the dataset and repeated, in-depth reading of all activity logs and reflective records. RO conducted a comprehensive, iterative analysis of the full dataset to achieve immersion and accurately capture patterns in CN practice. Additionally, AY reviewed the coding process, thematic structure, and conceptual interpretation. Through ongoing dialogue between RO and AY, themes were critically examined and refined, enhancing the credibility of the findings. Dependability was maintained by documenting the analytical process, including coding decisions, theme development, and interpretive steps. To enhance transparency, an audit trail was maintained throughout the analysis. This included records of episode extraction decisions, analytic memos documenting reflections on code meanings, iterative coding tables, theme development notes, and records of discussions between RO and AY regarding theme refinement and ICF linkage. Representative examples from the activity logs were retained for each major theme to ensure that the analytic interpretations could be traced back to the original data. Confirmability was ensured by grounding interpretations in the original activity logs and reflective practice records, rather than relying on the researcher's assumptions. RO conducted initial coding inductively based on the data, and AY provided independent feedback to challenge and refine interpretations. This collaborative analytic process helped minimize individual bias and ensured that findings reflected the data rather than preconceived perspectives. Transferability was supported through detailed descriptions of the study setting, CN roles, and community context, enabling readers to assess the applicability of findings to other community-based healthcare and rehabilitation settings.

Reflexivity was actively considered throughout the research process. RO is a physician and qualitative researcher with expertise in community-based healthcare, social medicine, and participation-oriented care. Although RO was not directly involved in the daily CN activities in Sarabetsu Village, RO's background in community health and participation-based care informed the interpretation of participation-related practices. To minimize potential interpretive bias, RO engaged in reflexive awareness and grounded interpretations strictly in the documented activity records. AY has extensive experience in CN education, training, and organizational management and has played a leadership role in developing CN practices. AY's practice-based expertise provided essential contextual insight into CN roles, relational practices, and organizational dynamics. At the same time, AY critically reviewed the analytical process to ensure that interpretations remained faithful to the documented data rather than reflecting assumptions based solely on professional experience. By integrating analytical rigor, systematic procedures, and reflexive collaboration among researchers with complementary expertise, this study ensured a robust, trustworthy qualitative analysis of CN practices and participation-oriented community engagement.

This study is part of a broader qualitative research project examining CN practices that facilitate participation in a rural Japanese community. The present analysis focuses specifically on the reconstruction of participation. Other analyses using the same dataset explore the roles of personal interests and environmental changes in enabling participation.

Ethical considerations

This study used existing activity records that were originally documented as part of routine CN practice. These records were subsequently used for secondary qualitative analysis as part of a broader research project examining CN practices. All identifiable personal information was anonymized prior to analysis to protect the privacy and confidentiality of community members. No personally identifiable information was included in the analytical dataset. This study was conducted in accordance with the ethical principles outlined in the Declaration of Helsinki and relevant ethical guidelines for research involving human-related data. Ethical approval for this study was obtained from the Ethics Committee of Unnan City Hospital (approval number: 20250005). The ethics committee approved the retrospective use of anonymized activity records for research purposes. As the study involved secondary analysis of anonymized records and did not involve direct intervention or interaction with participants, the ethics committee waived the requirement for individual informed consent.

## Results

Overview of the activity logs: participation as an everyday, "doable" practice

During the 12-month study period (January to December 2025), three CNs conducted a wide range of community-based activities to facilitate residents' engagement in social and community life. Analysis of activity logs and reflective practice records identified multiple types of participation-oriented activities occurring across diverse community settings.

CN activities were conducted in both formal and informal community settings, including community gathering spaces (e.g., Midori-no-ie), public facilities, cafés, hot spring waiting areas, and residents' homes. These activities involved direct interaction with residents, facilitating social activities, coordinating community events, supporting digital inclusion, and creating opportunities for interpersonal connection.

The activities addressed multiple dimensions of participation as defined by the ICF, including interpersonal interactions, community involvement, meaningful role engagement, and access to social resources. Importantly, participation was not limited to structured programs; it also emerged through everyday interactions, spontaneous conversations, and resident-led initiatives supported by CNs. Table 1 summarizes the major categories of CN activities, their objectives, and their relationship to participation within the ICF framework (Table 1).

**Table 1:** Summary of CNs' activities facilitating participation CNs: community nurses; ICF: International Classification of Functioning, Disability and Health

Activity category	Description of CN involvement	Community setting	Participation domain (ICF)	Representative examples from activity logs
Facilitation of community gathering spaces	CNs created and maintained safe and welcoming environments where residents could gather and interact	Community spaces (Midori-no-ie, hot spring lobby)	Community life, interpersonal interactions	Residents regularly gathered for conversation, creating ongoing social connections and informal support networks
Support for resident-led activities	CNs encouraged residents to initiate and sustain activities based on their interests and strengths	Community centers, cafés	Major life areas, community life	Residents organized mahjong sessions, community cafés, and creative activities with CN support
Role facilitation and empowerment	CNs supported residents in taking active roles within community activities	Community events, group activities	Major life areas, social roles	Residents served as instructors, facilitators, or event organizers
Narrative engagement and identity reconstruction	CNs engaged residents in conversations about their life experiences and interests, linking these to community participation	Multiple community locations	Interpersonal interactions, community life	Residents shared personal histories and hobbies, strengthening identity and social engagement
Digital participation support	CNs supported residents in using digital tools necessary for participation in modern community life	Community centers, public facilities	Communication, community life	Residents learned to use smartphone applications and digital services
Bridging community resources and individuals	CNs connected residents with community resources, people, and opportunities	Community-wide	Community life	CNs introduced residents to others, facilitated participation in events, and coordinated resources

To further characterize the scope and distribution of participation-oriented practices, participation episodes identified from the activity logs were categorized and quantified. A participation episode was defined as a documented interaction or activity in which CNs facilitated, enabled, or expanded residents' involvement in community life situations. Participation episodes facilitated by CNs spanned multiple domains of the ICF participation component, including community life (d910), recreation and leisure (d920), informal social relationships (d750), and communication using digital devices (d360). The most frequent participation episodes involved facilitating community gathering spaces and resident-led activities, highlighting the importance of relational environments and resident empowerment in enabling participation. These findings demonstrate that CNs operationalized participation across diverse ICF domains through everyday relational and community-based practices. Participation-oriented activities were observed consistently throughout the 12-month period (Table 2).

**Table 2:** Distribution of participation episodes facilitated by CNs and their linkage to ICF participation domains CNs: community nurses; ICF: International Classification of Functioning, Disability and Health

Activity category	ICF participation domain and code	Description	Number of participation episodes (n)	Percentage (%)
Facilitation of community gathering spaces	Community life (d910); informal social relationships (d750)	Participation in community gathering spaces such as Midori-no-ie and the hot spring lobby	34	25.4
Support for resident-led activities	Recreation and leisure (d920); community life (d910)	Mahjong sessions, community cafés, resident-led events	29	21.6
Role facilitation and empowerment	Remunerative employment (role-taking conceptually) (d850); informal social relationships (d750)	Residents serving as instructors, facilitators, or event coordinators	18	13.4
Narrative engagement and identity reconstruction	Basic interpersonal interactions (d710); informal social relationships (d750)	Storytelling, haiku creation, sharing personal experiences	22	16.4
Digital participation support	Communication devices and techniques (d360); recreation and leisure (d920)	Smartphone use, LINE communication, digital participation	14	10.4
Bridging community resources and individuals	Community life (d910); complex interpersonal interactions (d720)	Connecting residents to people, places, and community opportunities	17	12.7

Qualitative analysis of participation reconstruction facilitated by CNs

To further understand how CNs facilitated participation in community life, qualitative thematic analysis was conducted on activity logs and reflective practice records. This analysis identified recurring patterns in how participation was enabled, supported, and sustained within everyday community contexts.

Thematic analysis revealed that CNs facilitated participation not only by organizing or supporting activities but also by reconstructing participation as a relational, dynamic, and context-dependent process. Participation was enabled through the creation of accessible social environments, the facilitation of meaningful roles, the strengthening of interpersonal relationships, and the bridging of structural and technological barriers.

Four major themes were identified that describe how CNs operationalized participation within the ICF framework: (1) creating participation-ready environments, (2) facilitating reciprocal roles and resident empowerment, (3) reconstructing participation through personal narratives and identity, and (4) bridging participation barriers through relational and structural support.

These themes illustrate that participation was not a fixed outcome, but an evolving process shaped through ongoing interactions between CNs, residents, and the community environment. CNs functioned as relational facilitators who enabled residents to engage in meaningful life situations across multiple ICF participation domains (Table 3).

**Table 3:** Themes describing how CNs facilitated participation and their linkage to ICF participation domains CNs: community nurses; ICF: International Classification of Functioning, Disability and Health

Theme	Description	CNs practices	ICF participation domains and codes	Representative example from activity logs
Creating participation-ready environments	CNs created accessible, welcoming, and psychologically safe environments that enabled residents to engage in community life	Maintaining community gathering spaces, inviting residents to participate, facilitating informal interactions	Community life (d910); informal social relationships (d750)	Residents regularly gathered at Midori-no-ie and the hot spring lobby, forming stable participation environments
Facilitating reciprocal roles and resident empowerment	CNs enabled residents to take active roles in community activities, transforming them from recipients into contributors	Supporting residents to act as instructors, facilitators, and organizers of community activities	Recreation and leisure (d920); informal social relationships (d750)	Residents taught mahjong and facilitated community café activities
Reconstructing participation through personal narratives and identity	Participation was facilitated by connecting residents' life experiences, interests, and personal identities with community engagement opportunities	Engaging in conversations about personal history, facilitating creative activities such as haiku	Basic interpersonal interactions (d710); community life (d910)	Residents shared personal experiences and participated in haiku creation and storytelling
Bridging participation barriers through relational and structural support	CNs supported residents in overcoming barriers such as digital exclusion and lack of social access	Providing smartphone support, connecting residents with people and community resources	Communication devices and techniques (d360); community life (d910)	Residents learned to use smartphones and digital tools, enabling participation in community life

CNs created "participation-ready" spaces by redesigning micro-social conditions (invitation, accessibility, and psychological safety)

A recurring pattern was CNs' deliberate shaping of micro-environments to make participation socially safe and physically manageable. For example, in the hot spring lobby, participation was sustained by everyday "infrastructure" created jointly by residents: seating was flexibly expanded so that newcomers could join without disruption, and conversation flowed in a way that allowed people to enter and exit naturally. One participant explicitly described why the round table mattered: it allowed more chairs to be added so "everyone can talk together," and people routinely prepared seats for others when they arrived, turning a casual waiting space into a stable participation hub.

CNs also enacted participation through "small invitations" that respected a person's pace. In Midori-no-ie, a resident who initially declined ("it's already evening") was gently encouraged to stop by; after a short stay, the resident described the value of "dropping in" and leaving with renewed satisfaction, highlighting how brief, low-pressure interaction itself constituted meaningful participation.

Importantly, these spaces supported participation even for people with long-standing disengagement. In one vignette, a participant reflected that they had lived for decades without enjoyment, but that meeting CNs made weekly contact a meaningful anchor and something to look forward to, suggesting that participation was reconstructed not only as attendance but as restored anticipation and relational continuity.

Participation was reconstructed through reciprocity and role-making: residents became co-facilitators, not "recipients"

A consistent mechanism by which CNs strengthened participation was converting residents' strengths into visible roles and reciprocal contributions. This reduced the asymmetry of "supporter vs supported" and repositioned community members as capable actors within shared activities.

Peer Leadership and "Teaching Roles" Within Leisure Activities

In the mahjong activities, participation extended beyond playing; it included teaching, asking questions, and negotiating rules across tables. During a women's table session, an experienced participant served as an instructor, and when she left, others explicitly expressed how her presence enabled their engagement ("when he's gone, I suddenly don't understand"), while another participant actively signaled for help and asked questions, demonstrating both dependence and agency within a supportive learning climate.

Earlier in the year, residents themselves articulated the need to design participation formats for newcomers; one participant wished to organize "mahjong for people who don't understand mahjong," including the idea of teaching while playing, and also anticipated participation barriers (e.g., hearing difficulty) and the need for a supportive companion to prevent exclusion at a first experience. This shows participation being reconstructed as inclusive design work led by residents, with CNs enabling coordination.

Distributed Roles in Community Events (Preparation, Hosting, and Sustaining Continuity)

Participation expanded through role distribution in events such as community cafés. For example, a resident preparing to act as a "master substitute" requested instructions via social media, expressed anxiety about trying something new, and then carefully completed the hosting role. Positive feedback from visitors was relayed to him, reinforcing role confidence and legitimizing the resident's contribution to the social space.

Similarly, residents contributed assets (tools, supplies, labor) to enable events. In a café with traditional games and mochi-making, local supporters filled gaps by providing materials and know-how, emphasizing that households "have plenty" of what is needed and encouraging the team not to hesitate, turning private household resources into community participation assets.

A later café episode demonstrated the same logic through shared cooking: a resident anticipated the monthly rhythm of preparation and coordinated the recruitment of helpers, and the group proceeded efficiently through role-sharing and iterative problem-solving. This illustrates participation as ongoing co-production and succession planning rather than one-off attendance.

CNs connected personal narratives to meaningful "life situations," converting interests and histories into participation pathways

CNs frequently elicited residents' memories, preferences, and identities and then linked these to concrete participation opportunities, supporting "involvement in life situations" as personally meaningful roles rather than generic social activity.

In haiku sessions, participation was scaffolded as a creative practice grounded in lived experience. A participant selected seasonal words by imagining the coming week and using a season-word dictionary; the process involved iterative discussion and decision-making. Participation here was not simply writing haiku, but co-constructing meaning through shared language, seasonal imagination, and learning.

A second haiku vignette showed how participation expanded to include newcomers (an intern). The resident welcomed the young visitor with questions, connected the intern's travel experience to a seasonal theme ("spring journey"), and normalized that "bus, train, anything is fine," making the intern's lived experience legitimate material for participation. This reflects CNs enabling intergenerational participation through narrative reframing and the inclusive interpretation of "what counts." 

Likewise, residents' hobby knowledge (e.g., cultivation) became a participation anchor. In a café conversation about hydroponics, a resident explained that the practice began after surgeries and a hospital encounter with a farmer and described iterative experimentation (choosing uncommon crops, learning through failure and success). CNs treated this as valued expertise, reinforcing identity ("your greenhouse changes every time; your mind is working full speed"), which supported the resident's continued involvement and made their practice a resource for others' engagement (e.g., another resident expressing desire to visit and learn).

CNs expanded participation by bridging new participation barriers, especially digitalization, into shared, confidence-building learning

As community systems digitized, CNs' participation work increasingly included "digital participation": helping residents maintain involvement in village life when apps, messaging, and digital stamps became necessary for everyday functions.

One illustrative case involved a resident who repeatedly asked a family member for smartphone help and worried about burdening them. The resident attended a "smartphone chat session" with notes to avoid forgetting; peers and CN-linked supporters reframed learning as manageable ("decide to learn just one thing today"), proposed assistive tools (touch pen, phone case), and explicitly recognized the resident's existing strengths (mahjong ability) in a reciprocal manner. This repositioned the resident as a capable community member rather than a passive learner, thereby reconstructing participation as mutual respect and shared learning. 

Earlier logs also showed participation being supported through digital tools in everyday contexts. For example, a resident who had just registered a digital stamp immediately initiated a real-world trial at a local store and successfully completed it, despite previously claiming that older people "cannot do it." This vignette illustrates how CN support turned a potentially excluding digital requirement into an empowering experience that reinforced participation in ordinary community life (shopping, social joking, and shared stories during travel).

Synthesis: how CNs "reconstructed participation" in ICF terms

**Figure 1:**
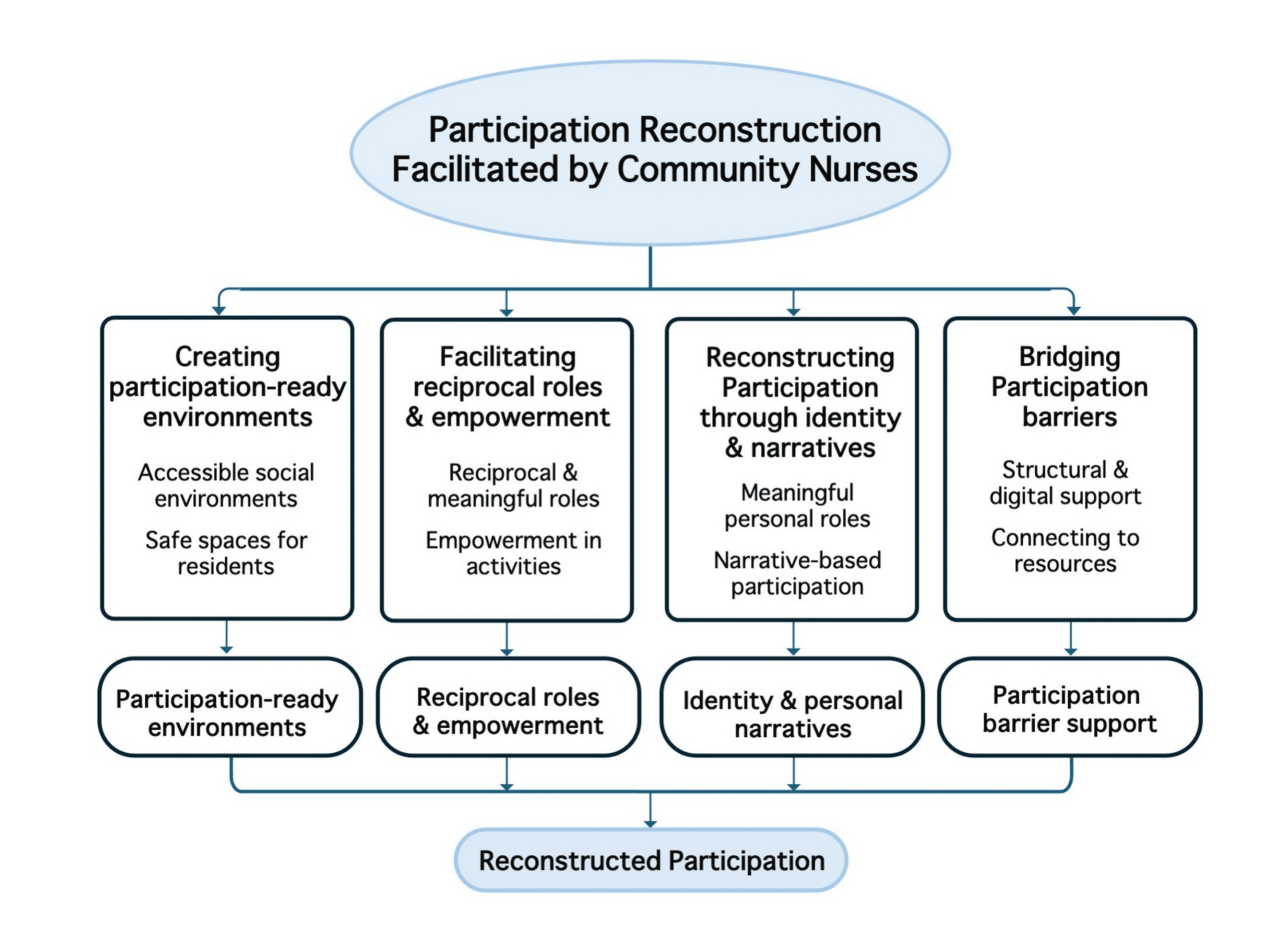
Themes describing how community nurses facilitated participation This figure presents the four themes identified through thematic analysis, illustrating the mechanisms by which community nurses facilitated participation in community life. Image created by Ryuichi Ohta using Microsoft PowerPoint (Microsoft Corp., Redmond, WA, USA)

Across themes, CNs did not treat participation as a discrete outcome (e.g., event attendance). Instead, they reconstructed participation as (1) accessible social micro-environments that invite entry and reduce risk, (2) reciprocal role-making that shifts residents into co-facilitators, (3) meaningful life linkage that converts narratives and skills into valued community roles, and (4) barrier-bridging participation, including digital inclusion, so that systemic changes do not produce new exclusion. Taken together, CNs' practices operationalized ICF participation as a relational, distributed, and continually renegotiated process within everyday rural community life (Figure 1).

## Discussion

Summary of the study

This study explored how CNs facilitated participation in community life by qualitatively analyzing activity logs and reflective practice records collected over a 12-month period in a rural Japanese community. Using thematic analysis informed by the ICF, this study identified four interrelated themes describing the mechanisms through which participation was enabled: creating participation-ready environments, facilitating reciprocal roles and empowerment, reconstructing participation through identity and narratives, and bridging participation barriers.

These findings demonstrate that CNs did not simply support participation through structured programs but actively reconstructed participation as a relational and dynamic process embedded in everyday community life. Participation was facilitated through the creation of socially and psychologically accessible environments, the redistribution of meaningful social roles, the integration of residents' personal identities into community engagement, and the reduction of structural and technological barriers.

Importantly, this study provides empirical evidence that CNs operationalized the participation component of the ICF in real-world community settings. While the ICF conceptualizes participation as involvement in life situations, this study illustrates how participation is actively constructed through relational facilitation, environmental adaptation, and ongoing interpersonal engagement.

Comparison with other studies

Previous studies have emphasized the importance of social participation as a determinant of health, particularly in aging populations [17,18]. Reduced participation has been associated with increased risks of functional decline, social isolation, cognitive impairment, and mortality [19]. Community-based interventions aimed at enhancing participation have therefore become an important component of health promotion and rehabilitation.

CNs have traditionally been described as providers of clinical care, case management, and coordination of health services [11,20]. However, emerging research has highlighted their role in promoting community engagement and addressing social determinants of health [21,22]. The present study extends this literature by demonstrating that CNs function not only as care providers but also as relational facilitators who actively reconstruct participation within community contexts.

Unlike conventional rehabilitation models, which often focus on restoring individual physical or cognitive function, this study's findings support a broader conceptualization of rehabilitation aligned with the ICF framework. Participation was not merely an outcome of improved function but was actively enabled through environmental modification, relational support, and role facilitation. This aligns with ecological and social models of rehabilitation, which emphasize the interaction between individuals and their social environments [23].

Furthermore, this study contributes to the growing literature on community-based health interventions by providing detailed qualitative evidence of how participation is facilitated in everyday practice. While previous studies have identified the importance of participation, fewer have examined the mechanisms by which healthcare professionals operationalize participation in community settings [24,25]. This study addresses this gap by identifying specific relational and environmental practices that enable participation.

Strengths of the study

This study has several important strengths. First, it used longitudinal activity logs and reflective practice records collected over a full year, allowing the detailed examination of participation facilitation across diverse contexts and over time. This provided rich, naturalistic data reflecting real-world CN practice rather than controlled or experimental interventions. Second, this study applied the ICF framework to analyze participation, providing a theoretically grounded interpretation of CN practices. By linking qualitative findings to ICF participation domains, this study bridges conceptual theory and real-world practice, contributing to a more operational understanding of participation. Third, the study employed rigorous qualitative methods, including systematic thematic analysis, iterative coding, and collaborative review by researchers with complementary expertise in qualitative research and CN practice. This enhanced the credibility and trustworthiness of the findings. Finally, this study offers practice-based insights into the role of CNs as facilitators of participation rather than solely providers of clinical care. These findings broaden the understanding of how CN activities may support participation-oriented community practice, while further research is needed to examine their broader relevance across settings and their relationship to resident-level outcomes.

Limitations of the study

This study has several limitations. First, the study was conducted in a single rural community in Japan, which may limit the transferability of findings to other settings, particularly urban or culturally different contexts. However, the mechanisms identified in this study may be applicable to other communities facing similar challenges related to aging and social isolation. Second, the study relied on activity logs and reflective records rather than direct observation or interviews with residents. While these records provide the detailed documentation of CN practices, they reflect not only what CNs observed in community settings but also how CNs professionally framed, prioritized, and interpreted participation processes. As a result, the findings may capture participation as documented through CNs' intentions, expectations, and practice orientations, rather than representing residents' experiences directly or exhaustively. Third, the quantification of participation episodes was used for descriptive mapping only and should not be interpreted as a measure of the frequency, intensity, quality, or effectiveness of participation outcomes. These counts indicate how often particular types of participation-facilitating practices were documented in the records, but they do not show how strongly participation was experienced, whether it was sustained over time, or whether it produced measurable benefits for residents. The primary purpose of this study was qualitative exploration rather than quantitative evaluation of impact. Finally, the researchers' positions may also have shaped the interpretation of the data. In particular, the close involvement of the research team in community-based practice and in understanding or supporting the development of CN activities may have influenced which aspects of participation were considered meaningful or analytically salient. Although reflexive analysis and collaborative review were used to critically examine interpretations, such procedures do not eliminate the possibility that the researchers' commitments and familiarity with the field shaped the analytic lens.

Relationship to broader research project

This study represents one component of a broader qualitative research project examining CN practices that facilitate participation in rural communities. While the present analysis focuses specifically on how participation is reconstructed through CN activities, other analyses derived from the same dataset explore related mechanisms, including the role of personal interests and environmental factors in enabling participation. Together, these complementary analyses aim to provide a more comprehensive understanding of participation-oriented community rehabilitation.

## Conclusions

This study demonstrates that CNs play a critical role in facilitating and reconstructing participation in community life. Through relational facilitation, environmental modification, role support, and barrier reduction, CNs operationalized participation as defined in the ICF framework. Participation was not simply an outcome but an ongoing relational process actively constructed within community contexts. These findings suggest that CNs can serve as key facilitators of participation-oriented rehabilitation and community health promotion. As aging populations increase worldwide, healthcare systems must move beyond function-focused models toward participation-oriented approaches. CNs are uniquely positioned to support this transition by enabling individuals to engage in meaningful life situations. Future research should further examine the impact of participation-oriented CN practices on health outcomes, quality of life, and community sustainability across diverse settings.
